# Association between Serum Uric Acid and Elevated Alanine Aminotransferase in the General Population

**DOI:** 10.3390/ijerph13090841

**Published:** 2016-08-24

**Authors:** Shuang Chen, Xiaofan Guo, Shasha Yu, Guozhe Sun, Hongmei Yang, Zhao Li, Yingxian Sun

**Affiliations:** Department of Cardiology, The First Affiliated Hospital of China Medical University, Shenyang 110000, China; loscs@126.com (S.C.); guoxiaofan1986@foxmail.com (X.G.); yidasasa@foxmail.com (S.Y.); gzhsun66@163.com (G.S.); eileen8222@163.com (H.Y.); meilichian@aliyun.com.cn (Z.L.)

**Keywords:** alanine aminotransferase (ALT), hyperuricemia, serum alanine aminotransferase, serum uric acid

## Abstract

Background: Both the serum uric acid (SUA) level and elevated alanine aminotransferase (ALT) are related to metabolic syndrome. However, the association between SUA and elevated ALT has not been elucidated in the general population. The objective of this study was to investigate the association between SUA and elevated ALT in the general population of China; Methods: A total of 11,572 adults (≥35 years of age) participated in this survey. Elevated ALT was defined as >40 U/L. SUA ≥ 7.0 mg/dL in males or ≥6.0 mg/dL in females was defined as hyperuricemia. SUA within the reference range was divided into quartiles, and its associations with elevated ALT were evaluated by logistic regressions; Results: A total of 7.4% participants had elevated ALT. The prevalence of hyperuricemia was 14.9% in males and 7.3% in females. There was a significantly positive dose-response association between SUA levels and the prevalence of elevated ALT. After adjusting for potential confounders, a positive relationship for elevated ALT was observed in subjects with hyperuricemia (odds ratio [OR]: 2.032, 95% confidence interval [CI]: 1.443–2.861 for men; OR: 2.045, 95% CI: 1.221–3.425 for women, both *p* < 0.05). Within the reference range, the association between SUA and elevated ALT persisted in the fourth quartile (OR: 1.467, 95% CI: 1.063–2.025 for men; OR: 1.721, 95% CI: 1.146–2.585 for women, both *p* < 0.05); Conclusions: Our results indicated that an increased SUA level, even within the reference range, was independently associated with elevated ALT in Chinese adults.

## 1. Introduction

Serum alanine aminotransferase (ALT) is a common liver enzyme of liver function tests and a sensitive indicator of hepatocyte injury [1,2,3]. It has been reported that elevated ALT is related to a range of health outcomes, such as metabolic disorders and cardiovascular diseases (CVD) [4]. Nonalcoholic fatty liver disease (NAFLD) is recognized as a leading cause of abnormal liver tests, and is characterized by fat accumulation in the liver. Because ALT is closely associated with fatty deposition in the hepatocytes, it is also commonly considered as a surrogate marker for NAFLD in some epidemiological studies [5,6]. Several studies have indicated that NAFLD is strongly associated with obesity, dyslipidemia, metabolic syndrome (MetS), diabetes mellitus, as well as cardiovascular events [4,7]. Thus, NAFLD is considered to be a hepatic consequence of metabolic diseases [8,9,10]. Recently, our studies had reported that there was a significant relationship between elevated ALT and cardiometabolic risk factors [11], and the serum ALT level, even within the reference range, was significantly associated with MetS [12].

Serum uric acid (SUA) is the major final product of the purine metabolism, and the level of SUA is maintained by the balance between SUA production and excretion [13]. Previous studies showed that SUA was an independent risk factor for CVD and MetS [14,15]. Furthermore, increasing evidence has suggested that not only hyperuricemia but also SUA within the reference range showed a positive correlation with MetS [15]. The SUA level was found to be increased in most NAFLD patients [7], which was an independent risk factor for NAFLD [16,17,18]. In fact, data had confirmed the strong relationship between NAFLD and MetS. Moreover, elevated SUA, although within the reference range, was clearly a component of MetS [19]. Therefore, increased SUA may play the role of linking NAFLD with MetS. Until now, scant studies among the general population have reported the relationship between SUA within the normal range and NAFLD.

To the best of our knowledge, the association between hyperuricemia and elevated ALT has not been evaluated in general adults, and only one study investigated the association between levels of SUA and elevated ALT in Israel [20], in which the authors did not define hyperuricemia or assess its relationship with elevated ALT. Therefore, hyperuricemia and SUA within the reference range need to be reviewed fully in relation to elevated ALT. Accordingly, we conducted this population-based cross-sectional study to investigate (1) the association between hyperuricemia with the morbidity of elevated ALT in a large-scale Chinese population; and (2) the relationship between normal SUA levels and elevated ALT in this representative population.

## 2. Materials and Methods

### 2.1. Study Population

We conducted a cross-sectional study from July 2012 to August 2013 in rural areas of Liaoning Province, which is called Northeast China Rural Cardiovascular Health Study (NCRCHS). A representative sample aged ≥35 years was selected to describe the prevalence, incidence and natural history of cardiovascular risk factors. The study adopted a multi-stage, stratified random cluster-sampling scheme. In the first stage of sampling, three counties (Zhangwu, Dawa, and Liaoyang County) were randomly selected to represent south, east and north of Liaoning Province. In the second stage, one town was randomly selected from each county (a total of three towns). In the third stage, eight to 10 rural villages were randomly selected from each township. In total, 26 rural villages were finally included. All eligible permanent residents aged ≥35 years from each village were selected for participation (a total of 14,016 participants); 11,956 individuals agreed and completed this cross-sectional study and the response rate was 85.3%. Approval for the NCRCHS was obtained from the Ethics Committee of China Medical University (Shenyang, China) (AF-SDP-07-1, 0-01). All participants provided written informed consent and all procedures were performed in accordance with the ethical standards. If the participants were illiterate, their proxies wrote the informed consents for them. In this study, we used data of baseline and only participants with complete data were included. After excluding 384 participants with uncompleted data, we made a final sample size of 11,572 (5356 men and 6216 women).

### 2.2. Data Collection

Data were collected during a single clinic visit by cardiologists and trained nurses using a standard questionnaire by face-to-face interview. Before the survey was performed, we invited all eligible investigators to attend the organized training. The training contents included the purpose of this study, how to administer the questionnaire, the standard method of measurement, the importance of standardization, and the study procedures. A strict test was evaluated after this training, only those who scored perfectly on the test could become investigators. During data collection, our inspectors had further instructions and support.

Data on demographic characteristics, lifestyle risk factors, medical history, were obtained by interview with a standardized questionnaire. The questionnaire was designed by statistical experts and clinical specialists. There was a central steering committee with a subcommittee for quality control. The project management office of Liaoning Province will check randomly for 5% questionnaires, a total of 27 unqualified questionnaires will be re-investigated again, and if the investigator made the fake questionnaire, we will cancel the qualification of this investigator and abandon all of his or her questionnaires. Educational level was divided into primary school or below, middle school and high school or above. The smoking and alcohol consumption status were also surveyed. Smoking and alcohol status were assessed by two types of questions, “Have you ever smoked at least one cigarette per day for over six months/Have you ever taken alcohol at least twice a week for over a year?” and “Do you smoke/take alcohol now?” Respondents were defined as current smokers/drinkers (those who answered YES to both questions), former smokers/drinkers (those who answered YES to the first question and NO to the second one), and never smokers/drinkers (those who answered NO to both questions). Physical activity included occupational and leisure-time physical activity. Occupational and leisure-time physical activity were merged and regrouped into the following three categories: (1) low—subjects who reported light levels of both occupational and leisure-time physical activity; (2) moderate—subjects who reported moderate or high levels of either occupational or leisure-time physical activity and (3) high—subjects who reported a moderate or high level of both occupational and leisure-time physical activity.

### 2.3. Blood Pressure Measurements

According to American Heart Association protocol, blood pressure was measured three times in a sitting position at 2 min intervals after at least 5 min of rest in a quiet room with the use of an automatic electronic sphygmomanometer (HEM-741C; Omron, Tokyo, Japan). Two doctors checked the calibration of the Omron device using a standard mercury sphygmomanometer every month under the British Hypertension Society protocol [21]. The mean of three BP measurements was taken and used in all analyses.

### 2.4. Anthropometric Measurements

Standing height and weight were measured to the nearest 0.1 cm and 0.5 kg using a wall-mounted stadiometer and an automated balance. Waist circumference (WC) was measured at the minimum circumference between iliac crest and the rib cage in standing position at the end of normal expiration using a non-elastic tape (to the nearest 0.1 cm). The body mass index (BMI) was calculated using the formula weight (kg)/height^2^ (m^2^).

### 2.5. Biochemical Measurements

Fasting (12 h overnight) blood samples were collected by venepuncture in EDTA tubes. Plasma was subsequently separated and frozen at −20 °C within 1 h for testing at a central, certified laboratory after collection. Fasting plasma glucose (FPG), plasma total cholesterol (TC), triglycerides (TG), low-density lipoprotein cholesterol (LDL-C), high-density lipoprotein cholesterol (HDL-C), serum uric acid (SUA), serum ALT and other biochemical parameters were analyzed enzymatically on an Olympus AU640 auto analyzer (Olympus, Kobe, Japan). All laboratory equipment was calibrated and blinded duplicate samples were used.

### 2.6. Definitions

Elevated serum ALT level was defined as ALT > 40 U/L [22], indicating an abnormal biochemical function of the liver. In the present study, hyperuricemia was defined according to sex-specific SUA levels: SUA ≥ 7.0 mg/dL for men and ≥6.0 mg/dL for women [23,24].

### 2.7. Statistical Analysis

Continuous variables were expressed as mean values and standard deviation (SD), whereas categorical variables were described as frequencies and percentages. Since data were normally distributed, continuous variables were compared between normal SUA and hyperuricemia by using Analysis of Variance (ANOVA) test. χ^2^-test analyses were used to examine associations between the categorical variables. Quartiles of normal SUA were created. Logistic regression was used to estimate the odds ratios (ORs) and 95% CIs for elevated ALT after adjustment for age, race, BMI, WC, SBP, DBP, TC, TG, HDL-C, LDL-C, FPG, history of CVD and lifestyle factors (smoking, drinking, family income, education, and physical activity). Serum ALT level within reference range was divided into quartiles so that the numbers of subjects in the four categories were almost equal, and the lowest category was set as reference. We used the area under the receiver-operating characteristic curve (AUC) and 95% confidence intervals (CIs) to assess the discriminatory power of serum uric acid levels to assess the risk for elevated ALT level. All statistical analyses were performed using SPSS version 19.0 software (SPSS Inc., Chicago, IL, USA), and *p* < 0.05 indicated statistical significance.

## 3. Results

### 3.1. Subject Characteristics

Table 1 and Table 2 showed the baseline characteristics of participants according to SUA levels. The study population was divided into five groups in both sexes: one group for hyperuricemia (>7.0 mg/dL for men; >6.0 mg/dL for women), and four groups within the reference range according to SUA quartiles. The SUA quartiles were as follows: <4.5, 4.5–5.2, 5.2–5.9, and 5.9–7.0 mg/dL for men; <3.5, 3.5–4.1, 4.1–4.7, and 4.7–6.0 mg/dL for women. Serum ALT levels increased linearly with the increasing SUA levels (*p* for trend <0.001). WC, BMI, DBP, TC, TG, LDL-C, and FPG were significantly higher in both genders, while HDL-C was lower, among subjects with higher SUA levels (*p* for trend <0.05 for all). SBP showed a linear trend in relation to SUA levels only in men (*p* for trend =0.004). In both sexes, subjects with hyperuricemia were significantly more likely to have a history of CVD (*p* < 0.05 for all). In men, subjects with hyperuricemia or those in the upper quartile of SUA were significantly more likely to be younger and current drinkers, while in women, the subjects were older with increasing levels of SUA (*p* or *p* for trend <0.05 for all).

### 3.2. Prevalence of Elevated ALT Categorized by SUA Levels for both Sexes

**Figure 1:**
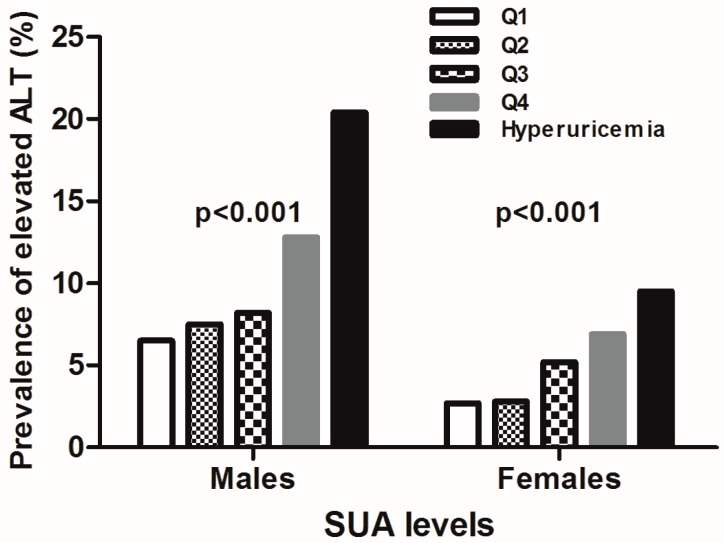
The proportion of adults with elevated ALT categorized by the uric acid level in both genders.

As shown in Figure 1, among both men and women, the prevalence of elevated ALT increased with an increment in the SUA level (*p* < 0.001). The proportions of elevated ALT from quartiles 1–4 of normal SUA to hyperuricemia were 6.5%, 7.5%, 8.2%, 12.8% and 20.4% for men, 2.7%, 2.8%, 5.2%, 6.9% and 9.5% for women, respectively. Among subjects in the fourth quartile and the hyperuricemia group, approximately 33.2% men and 16.4% women had elevated ALT. In addition, data showed that men had a higher prevalence of elevated ALT than women in all of the SUA categories.

### 3.3. Multivariate Analysis of the Independent Association between SUA and Elevated Serum ALT

**Figure 2:**
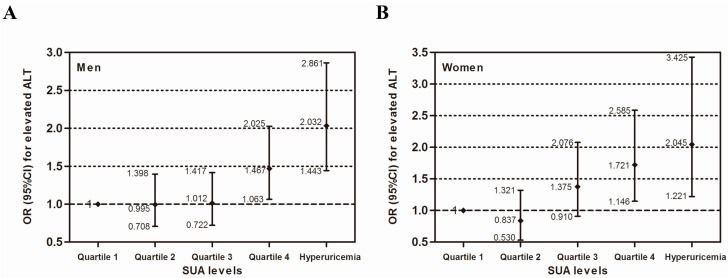
Multivariate analysis of the independent association between serum uric acid and elevated serum ALT by gender (**A**) for men and (**B**) for women. The model is adjusted for the same variables as in model 2 in Table 3.

Table 3 presents the multiple logistic regression analysis for elevated serum ALT. After adjusting for age and race in both genders (model 1), the logistic regression models showed that, comparing the highest SUA quartile and hyperuricemia with the lowest SUA quartile, SUA had a significant association with the elevated serum ALT. After further adjustment for WC, BMI, smoking, drinking, education level, physical activity, family income, history of CVD, BP, FPG and lipid profiles (model 2), we found that hyperuricemia was positively associated with elevated ALT (OR 2.032, 95% CI 1.443–2.861 for men; OR 2.045, 95% CI 1.221–3.425 for women). Subjects with hyperuricemia tended to exhibit a higher likelihood of having elevated ALT. Even within the reference range, subjects in the highest SUA quartile had a 1.467 times (for men) and 1.721 times (for women) higher risk of elevated ALT than those in the lowest SUA quartile (*p* < 0.05). The adjusted ORs and 95% CIs in all of the SUA categories for elevated ALT are shown in Figure 2.

### 3.4. The Area under the Receiver Operating Characteristic Curves (AUCs) (and 95% CIs) of Anthropometric Measures for the Presence of Elevated ALT

**Figure 3:**
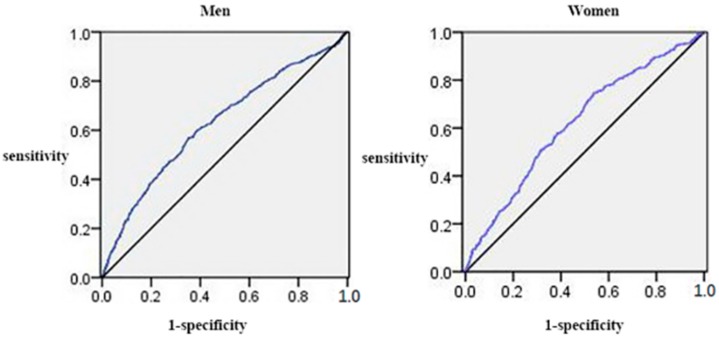
The discriminatory power of SUA in the prediction of elevated ALT. Area under the receiver operating characteristic curve of SUA was used to identify subjects with elevated ALT according to sex.

Elevated ALT was positively and significantly correlated with SUA in both genders (Figure 3). The discriminatory power of SUA in the prediction of elevated ALT was detected in both sexes (AUC: 0.627, 95% CI: 0.602–0.653 for men; AUC: 0.621, 95% CI: 0.588–0.653 for women).

## 4. Discussion

In our study, we showed that participants with hyperuricemia, even those in the highest quartile of SUA within the reference range, were more likely to have metabolic disorders and elevated ALT. This positive association between SUA and elevated ALT persisted after adjustment for possible confounding factors, both in men and women. Therefore, we suggested that more attention should be paid to the risk of elevated ALT in patients in the highest quartile of normal SUA or hyperuricemia.

During recent years, few studies have demonstrated that the SUA level was an independent risk factor for NAFLD [17,18]. Thus, SUA was recommended as a useful additional measure in the assessment of the risk for NAFLD in the clinical setting. Data from a Chinese study of 8925 employees showed that hyperuricemia was associated with NAFLD at baseline, and after a three-year follow up, SUA was positively related to the incidence of NAFLD [18,25]. Another study among obese adolescents found a significant association between hyperuricemia and elevated ALT [26]. Similar results of an association between SUA and NAFLD were also found in non-diabetic adults [16]. Moreover, Afzali A et al. supported the association between elevated SUA and the increased prevalence of chronic liver disease in a prospective study [27]. However, none of these studies reflected the association of both hyperuricemia and normal-range SUA with prevalence of elevated ALT in the general Chinese population. As a result, this study is the first population-based, cross-sectional survey to report this association in the general population of China.

Some previous studies have indicated that increased SUA, even within the normal range, was significantly correlated with the increased risk of CVD [28,29]. In addition, elevated SUA was demonstrated to be associated with hypertension and MetS [30]. SUA is the end product of the purine metabolism by the liver and hyperuricemia is a common feature in patients with MetS or its components, including increased triglycerides and obesity. It is noteworthy that SUA plays a powerful antioxidant role [31] which provides an antioxidant defense against oxidative stress [32,33]. It is known that oxidative stress is considered to be a marker of MetS and CVD [7]. In addition, our previous study has supported that elevated ALT is positively related to MetS [12]. Therefore, increased SUA might be correlated with elevated ALT. In another study conducted by Shira Zelber-Sagi et al. [20], SUA was independently associated with elevated ALT for the fourth quartile vs. the first and did not define hyperuricemia. However, this present study found that hyperuricemia was associated with elevated ALT, and a normal-high value of SUA was also positively correlated with elevated ALT, which proved our hypothesis.

Several potential mechanisms might be responsible for the positive association between increased SUA and elevated ALT. Hyperuricemia and NAFLD share similar metabolic disorders, such as diabetes, insulin resistance, central obesity, and dyslipedimia, which are all potential risk factors for NAFLD [34,35]. Insulin resistance not only led to hyperinsulinemia, which would cause a reduction of renal SUA excretion [36], but it also made hepatocytes more vulnerable to damage due to certain triggers, such as fat accumulation in the liver [37]. Another plausible explanation for the association between SUA and elevated ALT would be oxidative stress. In NAFLD patients, increased levels of SUA may alter the endogenous anti-oxidant defense against the peroxidation of liver fat, and therefore promote the progression of liver damage [38,39]. The present study indicated the positive associations of high-normal SUA and hyperuricemia with elevated ALT. Our data implied that increased SUA, even within the reference range, can trigger the clinical suspicion of elevated ALT. Thus, we suggest that SUA levels might be an important risk factor for elevated ALT.

Study limitations and strengths should be mentioned. Firstly, because of its cross-sectional design, we were unable to answer the question of whether a high-normal SUA level was a cause or an effect of elevated ALT. Further research in the future is needed to clarify their interrelationship. Secondly, despite extensive adjustment being done in our study, there are known causes of elevated ALT that were not tested in our study, such as chronic viral hepatitis, and the possibility still exists that unmeasured confounders may explain part of the association. However, the strengths of this study are its population-based design, large sample size, and the extensive information on confounders. Our study for the first time provided strong evidence that hyperuricemia and normal-high SUA play a crucial role in implying the increased risk for elevated ALT in the general population of China. It will be necessary to confirm the effect of the SUA levels on predicting elevated ALT in a future longitudinal study, and replication of the results in further investigations with a large-scale population are necessary before firm conclusions can be drawn.

## 5. Conclusions

In summary, in this study, increased serum uric acid (SUA) levels, even within the reference range, were independently correlated with the presence of elevated alanine aminotransferase (ALT). This finding implies that Chinese adults with hyperuricemia or a high-normal SUA level would be regarded as a group at increased risk for elevated ALT.

## Figures and Tables

**Table 1:** Characteristics of subjects according to serum uric acid level in men (*n* = 5356).

Variables	Quartiles of Normal SUA				Hyperuricemia	*p* or *p* for Trend
	Q1 (*n* = 1115)	Q2 (*n* = 1146)	Q3 (*n* = 1128)	Q4 (*n* = 1167)	(*n* = 800)	
SUA, mg/dL	3.9 ± 0.5	4.9 ± 0.2	5.5 ± 0.2	6.4 ± 0.3	8.0 ± 1.0	<0.001 *
ALT, U/L	22.7 ± 18.8	23.5 ± 17.9	24.4 ± 18.2	27.7 ± 31.9	31.6 ± 22.3	<0.001 *
Age, year	56.1 ± 10.4	55.0 ± 10.7	53.9 ± 10.8	53.4 ± 10.8	53.0 ± 11.0	<0.001 *
Race(Han), %	1051 (94.3)	1079 (94.2)	1070 (94.9)	1104 (94.6)	766 (95.8)	0.573
Current smokers, %	662 (59.4)	677 (59.1)	649 (57.5)	656 (56.2)	411 (51.4)	0.004 #
Current drinkers, %	434 (38.9)	499 (43.5)	501 (44.4)	595 (51.0)	404 (50.5)	<0.001 *
High school or above, %	97 (8.7)	128 (11.2)	136 (12.1)	143 (12.3)	109 (13.6)	0.006 #
Physical activity, %						0.015 #
High	59 (5.3)	72 (6.3)	67 (5.9)	67 (5.7)	32 (4.0)	
Moderate	796 (71.4)	844 (73.6)	810 (71.8)	851 (72.9)	550 (68.8)	
Low	260 (23.3)	230 (21.1)	251 (22.3)	249 (21.3)	218 (27.3)	
Family income > 20,000 CNY/y, %	314 (28.2)	354 (30.9)	388 (34.4)	434 (37.2)	277 (34.6)	<0.001 *
History of CVD, %	124 (11.4)	94 (8.4)	113 (10.3)	113 (9.8)	114 (14.5)	0.001 #
SBP, mm Hg	140.2 ± 22.4	140.7 ± 22.9	141.6 ± 22.2	141.8 ± 22.2	143.7 ± 22.5	0.004 #
DBP, mm Hg	81.2 ± 11.1	81.9 ± 11.9	82.4 ± 11.9	83.9 ± 12.2	86.4 ± 12.8	<0.001 *
WC, cm	80.5 ± 9.3	82.1 ± 9.1	83.5 ± 9.4	85.5 ± 9.5	88.9 ± 9.8	<0.001 *
BMI, kg/m^2^	23.8 ± 3.3	24.2 ± 3.3	24.6 ± 3.5	25.2 ± 3.4	26.3 ± 3.8	<0.001 *
TC, mmol/L	5.0 ± 0.9	5.1 ± 1.0	5.1 ± 1.0	5.2 ± 1.0	5.4 ± 1.1	<0.001 *
TG, mmol/L	1.2 ± 0.9	1.4 ± 1.1	1.6 ± 1.5	1.8 ± 1.8	2.4 ± 2.4	<0.001 *
HDL-C, mmol/L	1.5 ± 0.5	1.4 ± 0.4	1.4 ± 0.4	1.3 ± 0.4	1.1 ± 0.2	<0.001 *
LDL-C, mmol/L	2.8 ± 0.8	2.8 ± 0.8	2.9 ± 0.8	3.0 ± 0.8	3.2 ± 0.8	<0.001 *
FPG, mmol/L	5.9 ± 1.4	5.9 ± 1.5	5.9 ± 1.5	6.0 ± 1.4	6.1 ± 2.3	0.001 #

Notes: Data are expressed as the mean ± SD or as *n* (%). The significance of comparison of continuous variables across the SUA groups was shown by *p* for trend and *p* for categorical variables, respectively. Abbreviations: SUA, serum uric acid; ALT, alanine aminotransferase; CNY, China Yuan (1CNY = 0.161 USD); CVD, cardiovascular diseases; BMI, body mass index; WC, waist circumference; SBP, systolic blood pressure; DBP, diastolic blood pressure; TC, total cholesterol; TG, triglyceride; LDL-C, low-density lipoprotein cholesterol; HDL-C, high-density lipoprotein cholesterol; FPG, fasting plasma glucose. * *p* < 0.001, # *p* < 0.05.

**Table 2:** Characteristics of subjects according to serum uric acid level in women (*n* = 6216).

Variables	Quartiles of Normal SUA				Hyperuricemia	*p* or *p* for Trend
	Q1 (*n* = 1412)	Q2 (*n* = 1439)	Q3 (*n* = 1472)	Q4 (*n* = 1442)	(*n* = 451)	
SUA, mg/dL	3.0 ± 0.4	3.8 ± 0.2	4.4 ± 0.2	5.3 ± 0.3	6.8 ± 0.9	<0.001 *
ALT, U/L	17.7 ± 10.0	18.2 ± 11.7	20.2 ± 13.5	21.7 ± 15.4	23.0 ± 14.3	<0.001 *
Age, year	51.1 ± 9.8	52.2 ± 10.0	53.7 ± 10.3	55.1 ± 10.4	57.7 ± 10.5	<0.001 *
Race(Han), %	1312 (92.9)	1363 (94.7)	1396 (94.8)	1386 (96.1)	440 (97.6)	<0.001 *
Current smokers, %	224 (15.9)	225 (15.6)	247 (16.8)	255 (17.7)	79 (17.5)	0.549
Current drinkers, %	37 (2.6)	42 (2.9)	45 (3.1)	42 (2.9)	17 (3.8)	0.797
High school or above, %	101 (7.2)	126 (8.8)	105 (7.1)	113 (7.8)	35 (7.8)	<0.001 *
Physical activity, %						<0.001 *
High	56 (4.0)	85 (5.9)	85 (5.8)	98 (6.8)	32 (7.1)	
Moderate	890 (63.0)	874 (60.7)	842 (57.2)	793 (55.0)	229 (50.8)	
Low	466 (33.0)	480 (33.4)	545 (37.0)	551 (38.2)	190 (42.1)	
Family income > 20,000 CNY/y, %	466 (33.0)	488 (33.9)	497 (33.8)	472 (32.7)	136 (30.2)	0.210
History of CVD, %	211 (15.2)	234 (16.5)	274 (19.0)	300 (21.3)	135 (30.7)	<0.001 *
SBP, mm Hg	135.3 ± 23.7	134.9 ± 23.2	136.9 ± 23.3	140.5 ± 24.6	143.7 ± 24.4	0.202
DBP, mm Hg	78.0 ± 11.4	78.6 ± 11.5	79.6 ± 11.5	81.5 ± 12.0	82.9 ± 12.5	<0.001 *
WC, cm	78.1 ± 9.1	79.4 ± 9.1	81.4 ± 9.6	84.3 ± 9.3	87.1 ± 9.9	<0.001 *
BMI, kg/m^2^	23.9 ± 3.7	24.3 ± 3.6	24.8 ± 3.7	25.9 ± 3.7	26.6 ± 3.9	<0.001 *
TC, mmol/L	5.0 ± 1.1	5.2 ± 1.1	5.3 ± 1.1	5.5 ± 1.1	5.8 ± 1.4	<0.001 *
TG, mmol/L	1.2 ± 0.7	1.4 ± 1.0	1.6 ± 1.3	1.9 ± 1.5	2.5 ± 2.2	<0.001 *
HDL-C, mmol/L	1.5 ± 0.4	1.4 ± 0.3	1.4 ± 0.3	1.3 ± 0.3	1.3 ± 0.3	<0.001 *
LDL-C, mmol/L	2.8 ± 0.8	2.9 ± 0.8	3.0 ± 0.8	3.1 ± 0.8	3.3 ± 0.9	<0.001 *
FPG, mmol/L	5.8 ± 1.8	5.8 ± 1.7	5.8 ± 1.4	5.9 ± 1.5	6.2 ± 1.6	<0.001 *

Notes: Data are expressed as the mean ± SD or as *n* (%). The significance of comparison of continuous variables across the SUA groups was shown by *p* for trend and *p* for categorical variables, respectively. Abbreviations: SUA, serum uric acid; ALT, alanine aminotransferase; CNY, China Yuan (1 CNY = 0.161 USD); CVD, cardiovascular diseases; BMI, body mass index; WC, waist circumference; SBP, systolic blood pressure; DBP, diastolic blood pressure; TC, total cholesterol; TG, triglyceride; LDL-C, low-density lipoprotein cholesterol; HDL-C, high-density lipoprotein cholesterol; FPG, fasting plasma glucose. * *p* < 0.001.

**Table 3:** Multiple logistic regression for elevated ALT.

SUA	Model 1			Model 2		
**Males**	**OR**	**95% CI**	***p*-Value**	**OR**	**95% CI**	***p*-Value**
Quartile 1 (<4.5 mg/dL)	1	–	–	1	–	–
Quartile 2 (4.5–5.2 mg/dL)	1.101	0.795–1.525	0.563	0.995	0.708–1.398	0.976
Quartile 3 (5.2–5.9 mg/dL)	1.145	0.830–1.580	0.410	1.012	0.722–1.417	0.947
Quartile 4 (5.9–7.0 mg/dL)	1.865	1.387–2.508	<0.001 *	1.467	1.063–2.025	0.020 ^#^
Hyperuricemia (≥7.0 mg/dL)	3.236	2.405–4.355	<0.001 *	2.032	1.443–2.861	<0.001 *
**Females**						
Quartile 1 (<3.5 mg/dL)	1	–	–	1	–	–
Quartile 2 (3.5–4.1 mg/dL)	0.963	0.616–1.507	0.871	0.837	0.530–1.321	0.445
Quartile 3 (4.1–4.7 mg/dL)	1.906	1.289–2.818	0.001	1.375	0.910–2.076	0.130
Quartile 4 (4.7–6.0) mg/dL)	2.608	1.788–3.805	<0.001 *	1.721	1.146–2.585	0.009 ^#^
Hyperuricemia (≥6.0 mg/dL)	3.809	2.426–5.980	<0.001 *	2.045	1.221–3.425	0.007 ^#^

Notes: Abbreviations: SUA, serum uric acid; Model 1: Adjusted for age, race; Model 2: Adjusted for age, race, WC, BMI, smoking, drinking, education level, physical activity, family income, history of CVD, BP, FPG, serum creatinine and lipid profiles. * *p* < 0.001; ^#^ *p* < 0.05.

## Abbreviations

The following abbreviations are used in this manuscript:

SBP	Systolic blood pressure
DBP	Diastolic blood pressure
WC	Waist circumference
BMI	Body mass index
TC	Total cholesterol
TG	Triacylglycerol
HDL-C	High-density lipoprotein cholesterol
LDL-C	Low-density lipoprotein cholesterol
FPG	Fasting plasma glucose
ALT	Alanine aminotransferase
CVD	Cardiovascular diseases
NAFLD	Nonalcoholic fatty liver disease
MetS	Metabolic syndrome
SUA	Serum uric acid
AUC	Area under the receiver operating characteristic curve
